# *KIF21A* mutations in two Chinese families with congenital fibrosis of the extraocular muscles (CFEOM)

**Published:** 2010-10-13

**Authors:** Xian Yang, Koki Yamada, Bradley Katz, Hongzai Guan, Lifei Wang, Caroline Andrews, Guiqiu Zhao, Elizabeth C. Engle, Haoyu Chen, Zongzhong Tong, Jie Kong, Cong Hu, Qinglan Kong, Guiyun Fan, Ze Wang, Meizhen Ning, Shaoyan Zhang, Jinling Xu, Kang Zhang

**Affiliations:** 1 Department of Ophthalmology, Medical College of Qingdao University, the Affiliated Hospital of Medical College Qingdao University, Qingdao, China; 2 Program in Genomics,the Department of Medicine, Children’s Hospital Boston and Harvard Medical School, Boston, MA; 3 Department of Ophthalmology and Visual Sciences, Moran Eye Center, Salt Lake City, UT; 4 Program in Human Molecular Biology & Genetics, Eccles Institute of Human Genetics, University of Utah School of Medicine, Salt Lake City, UT; 5 Department of Neurology, Children’s Hospital Boston and Harvard Medical School, Boston, MA and the Howard Hughes Medical Institute, Chevy Chase MD; 6 Departments of Ophthalmology, FM Kirby Neurobiology Center, and The Manton Center for Orphan Disease Research, Children’s Hospital Boston, Harvard Medical School, Boston, MA; 7 Joint Shantou International Eye Center, Shantou University and the Chinese University of Hong Kong, Shantou, China; 8 Yellow Sea fisheries research institution, Qingdao, China; 9 Department of Ophthalmology, Xingtai Eye Hospital, Xingtai, China; 10 Department of Ophthalmology, Dongnan Eye Hospital, Nanjing, China; 11 Department of Hematology, Medical College of Qingdao University, Qingdao, China; 12 Clinical laboratory, the Affiliated Hospital of Medical College Qingdao University, Qingdao, China; 13 Department of Ophthalmology, Wenzhou Medical School, Wenzhou, China; 14 Institute for Genomic Medicine and Shiley Eye Center, University of California San Diego, San Diego, CA

## Abstract

**Purpose:**

Two Chinese families (XT and YT) with congenital fibrosis of the extraocular muscles (CFEOM) were identified. The purpose of this study was to determine if previously described Homo sapiens kinesin family member 21A (*KIF21A*) mutations were responsible for CFEOM in these two Chinese pedigrees.

**Methods:**

Clinical characterization and genetic studies were performed. Microsatellite genotyping for linkage to the CFEOM1 and CFEOM3 loci was performed. The probands were screened for *KIF21A* mutations by bidirectional direct sequencing. Once a mutation was detected in the proband, all other participating family members and 100 unrelated control normal individuals were screened for the mutation.

**Results:**

All affected individuals in family XT shared the common manifestations of CFEOM1. Family YT had two affected individuals, a mother and a daughter. The daughter had CFEOM1, while her mother never had congential ptosis but did have limited extraocular movements status post strabismus surgery. Haplotype analysis revealed that pedigree XT was linked to the 12q CFEOM1 locus and the affected memberes harbored the second most common missense mutation in *KIF21A* (2,861G>A, R954Q). Family YT harbored the most common missense de novo mutation in *KIF21A* (2,860C>T, R954W). Both of these mutations have been previously described.

**Conclusions:**

The observation of these two *KIF21A* mutations in a Chinese pedigree underscores the homogeneity of these mutations as a cause of CFEOM1 and CFEOM3 across ethnic divisions.

## Introduction

Congenital fibrosis of the extraocular muscles (CFEOM) is a hereditary ocular motility disorder characterized by non-progressive restrictive ophthalmoplegia and ptosis [1].Three inherited forms have been described: CFEOM Type 1 (CFEOM1; OMIM 135700) and CFEOM3 (OMIM 600638, 607034) are autosomal dominant; CFEOM2 (OMIM 602078) is autosomal recessive. Three genetic loci (fibrosis of the extraocular muscles 1 [FEOM1], FEOM2, and FEOM3) and 3 disease-causing genes (Homo sapiens kinesin family member 21A [*KIF21A*], paired-like homeobox 2a, [*ARIX* or *PHOX2A*], and tubulin beta 3, [*TUBB3*]) have been identified.

The most common form of CFEOM is CFEOM1 (“classic” CFEOM). Individuals have bilateral ophthalmoplegia and ptosis from birth. The primary vertical position of each eye is infraducted at least 10° with the inability to raise either eye above the horizontal midline and affected individuals demonstrate a characteristic “chin up” head position. Horizontal movement is also impaired, but the degree of residual horizontal movement can vary between affected individuals. Individuals frequently have amblyopia in at least one eye. CFEOM1 is inherited as a fully penetrant autosomal dominant trait and maps to the CFEOM1 locus on chromosome 12cen [2-5]. The disease-causing gene, *KIF21A,* belongs to a family of kinesin motor proteins. The most common *KIF21A* mutations alter one of several conserved amino acid residues within the *KIF21A* stalk region that are thought to interfere with *KIF21A* dimerization [6]. Post-mortem pathologic studies have shown that CFEOM1 is caused primarily by the absence or maldevelopment of the superior division of the oculomotor nerve or the corresponding subnuclei [7].

Individuals with the recessive disorder CFEOM2 were found in consanguineous pedigrees, mapped to the CFEOM2 locus on 11q13 [8] and result from homozygous mutations in *ARIX* (*PHOX2A*) [9,10]. *ARIX* encodes a homeodomain transcription factor essential to the development of the oculomotor and trochlear motoneurons in mice and zebrafish [11,12]. The CFEOM2 phenotype is characterized by bilateral exotropia, ophthalmoplegia, and ptosis from birth.

CFEOM3 is much less common than CFEOM1 and is inherited as an autosomal dominant trait with variable penetrance and variable expressivity. By definition, CFEOM3 families contain at least one affected member with unilateral disease, no ptosis, eyes that are not infraducted in primary eye position, or the ability to raise at least one eye above the horizontal midline [13]. Most families with CFEOM3 map to 16qter [14-16] and harbor *TUBB3* mutations [17], but some families map to the CFEOM1 locus and harbor *KIF21A* mutations [13-16].

We clinically characterized two Chinese families with autosomal dominant CFEOM. To determine if these two families were linked to the CFEOM1 or CFEOM3 loci, we performed linkage analysis. We then performed mutation screening of three coding exons of *KIF21A* (exon 8, 20, and 21) that contain the previously described *KIF21A* mutations.

## Methods

### Clinical studies

Ophthalmologic examinations were performed and blood samples were obtained from all participants after obtaining informed consent. Investigations were conducted according to the guidelines of the Declaration of Helsinki. The study was approved by and performed at the Affiliated Hospital of Medical College Qingdao University, Qingdao Municipal Science and Technology Commission.

The primary position of gaze, ductions, and versions with cover test were analyzed and quantified in 6 diagnostic positions. Affected individuals underwent forced duction testing. Globe retraction and/or aberrant movements were observed. The width of the palperbral fissure was measured. Levator function was measured from the upper lid margin while the individual attempted supraduction from the infraducted position without recruiting the frontalis muscle. Ptosis was defined if the upper lid covered 2 mm or more of the iris. Ptosis was graded as mild if the upper lid covered the iris above the upper papillary margin, moderate if it occluded up to half the pupil, and severe if it occluded more than half of the pupil [18]. Visual acuity and non-cycloplegic refraction were obtained when possible. All of the ophthalmologic examinations were recorder with a digital camera.

### Molecular genetic studies

#### Genotyping

Blood samples were obtained from all participating family members. Lymphocyte DNA was extracted using the Puregene kit (Waston’s biotechnique company, Shanghai, China). Linkage studies were conducted using the following polymorphic DNA microsatellite markers from the FEOM1 and FEOM3 regions: D12S345, D12S59, D12S331, and D12S1048 were analyzed to assess linkage to the dominant CFEOM1region; D16S3063, D16S689, D16S3026, and D16S3121 were analyzed to assess linkage to the dominant CFEOM3 region. The primer sequences are available from the Genome Data Base (Table 1). Primers were purchased from Biotechnologies Incorporation (Shanghai, China). Amplification was performed as reported elsewhere using standard techniques [3,14]. Briefly, each polymorphic DNA microsatellite marker was amplified for 35 cycles of 40 s at 94 °C for 75 s at the annealing temp as indicated in Table 1 for a given primer set, 20 s at 72 °C and followed by 7 min at 72 °C. The polymerase chain reaction products were separated on 6% denaturing polyacrylamide gels, and the alleles were visualized by silver staining.

**Table 1:** Primers for microsatellite markers.

**Marker**	**D number**	**Primer sequences (5’>3’)**	**Annealing temperature (°C)**	**Product size (bp)**
ATA29H01	D12S1048	GGTCTGCTTAGGTCCCTTTT	55	209-229
		AAGGAACCAAGGAGTGGAAG		
Mfd75A	D12S59	CTCACTCATGCTTGTTTTGA	60.3	164-179
		GATCACGTCAGACTGGGCT		
AFM092wd11	D12S331	TAACATTTATTCATCTCCATACTGA	57.8	175-181
		ACATGTAAGAGAGNAAGTTTACAAA		
AFM296yg5	D12S345	CAGCCTGGGTAACAAA	57.8	209-238
		AGCAATGTGTGCATTC		
UT8102	D16S689	GCCTGGGCGATAAGTGAGA	56	209
		TCACCACAACCCAAACATTC		
AFM156xb8	D16S3026	CTCCCTGAGCAACAAACACC	55	178-210
		GGTCATTTATATGCGCCTGA		
AFMa306yf1	D16S3063	AGCTTGTTTTAGCAANAATTTC	59	218-260
		TGCCTGATTCCACCAA		
AFM342xd9	D16S3121	CATGTTGTACATCGTGATGC	58	79-85
		AGCTTTTATTTCCCAGGGGT		

Lod scores were calculated for the family XT using the Linkage version 5.2 package of programs with the assumption of autosomal dominant inheritance with complete penetrance, a disease incidence of 1 in 1 million births, and 10 marker alleles of equal frequency, as previously described [4,18]. Haplotype analysis was examined in family YT to determine segregation of the potentially affected allele among family members.

#### KIF21A mutation detection

Mutation detection was conducted by PCR amplification of exons 8, 20, and 21 of the *KIF21A* gene and flanking intron-exon boundaries from genomic DNA of each proband in the two families. The PCR sequencing primers and conditions were listed in Table 2. Briefly, the procedure of PCR was as follows: Cycle 1: initial denaturation at 95 °C for 15 min. Cycle 2-15: touchdown PCR: denature at 94 °C × 40 s, anneal × 60 s (start annealing at 7 °C above the annealing temp as indicated in Table 2 for a given primer set and decrease in increments of 0.5 °C /cycle for a total of 14 cycles), elongate at 72 °C for 60 s. Cycles 16-35: 94 °C for 40 s, annealing temp as indicated in Table 2 for a given primer set for 60 s, 72 °C for 60 s. Cycle 36: final extension at 72 °C for 10 min [6]. The amplicons were subjected to bidirectional direct DNA sequencing on an ABI 3730 DNA sequencer (Applied Biosystems Inc., Carlsbad, CA). To further establish the pathogenicity of mutations detected in the probands, all participating family members and 100 unrelated, normal Chinese individuals were screened for the mutation by direct sequencing.

**Table 2:** *KIF21A *PCR primers.

**Exon**	**Primer sequences (5’>3’)**	**Product size (bp)**	**Annealing temperature (°C)**
21	F:GGGGGTTTGATGGTACTGTAT	354	54
	R:CTTCATGTAAAAACTGAAAGTGCT		
20	F:AAATGGCTCATTATTTGGCA	304	52
	R:CAGGGAACAAAATTGGAAGA		
8	F:TTTTAGCATTTTAGGTGCTTTT	306	52
	R:AAAGTGCCAGCCTTAGATGT		

Note: F:forward primers; R:reverse primers

## Results

### Clinical findings

**Figure 1:**
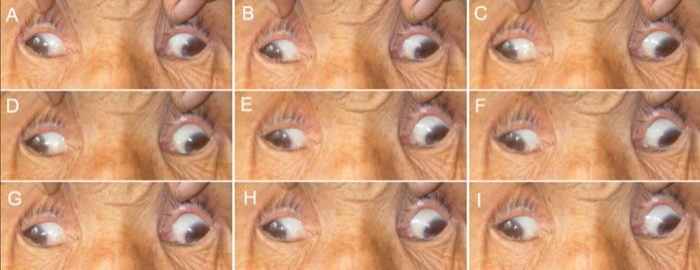
Photographs of individual II:4 in pedigree XT. Photos are taken in primary gaze (**E**) and in the 8 cardinal gaze positions (**A**, **B**, **C**, **D**, **F**, **G**, **H**, and **I**). This subject demonstrates marked ophthalmoparesis, infraducted eyes in primary position, exotropia, and an inability to raise the eyes above midline. This patient also has bilateral ptosis (not shown).

**Figure 2:**
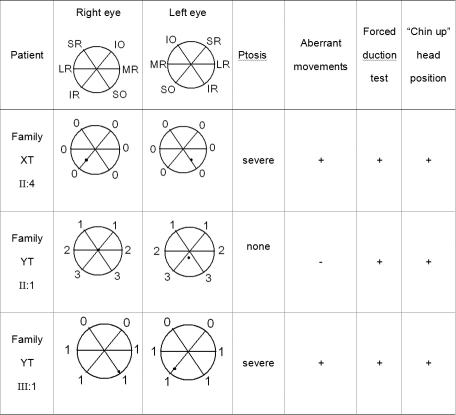
Clinical features of three individuals from two Chinese pedigrees with CFEOM. The second and third columns document the range of extraocular movement in each eye and the position of the eyes in primary position. Extraocular movement is rated on a scale of 0 to 4, with 0 indicating no movement in that direction and 4 indicating normal movement in that direction. The black dot indicates the position of the pupil within the orbit in primary gaze. SR=superior rectus; IR=inferior rectus; LR=lateral rectus; MR=medial rectus; SO=superior oblique; IO=inferior oblique. The presence of ptosis, aberrant eye movements, forced duction testing, and “chin-up” head position is also noted.

Family XT is from He Bei Province, China. All affected members shared the typical clinical features of CFEOM1 that have been reported previously in other ethnic families: congenital non-progressive ptosis, infraducted globe position in primary gaze, and upgaze and horizontal gaze palsy in both eyes. All patients including the proband, had evidence of aberrant innervation with nystagmoid movements in all directions of gaze. Forced duction testing of the superior rectus muscles and medial rectus muscles was positive (Figure 1 and Figure 2). Bell phenomenon was absent. Clinicopathological studies showed fibrotic extraocular muscles in the proband.

**Figure 3:**
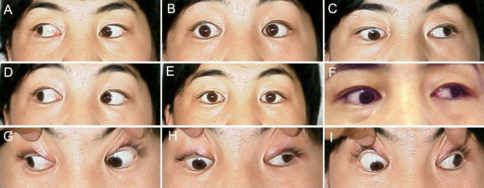
Motility photographs of individual II:1 from pedigree YT. Photos are taken in primary gaze (**E**) and in the 8 cardinal gaze positions (**A**, **B**, **C**, **D**, **F**, **G**, H, **I**). This individual had strabismus surgery at age 18 years and does not demonstrate all of the typical features of CFEOM. Although her upgaze is restricted, her eyes are not infraducted in primary position and she has no ptosis. Downgaze and horizontal eye movements are relatively well preserved. Her parents are clinically unaffected and her daughter has typical features of CFEOM1 (Figure 2). This individual has a de novo *KIF21A* mutation.

**Figure 4:**
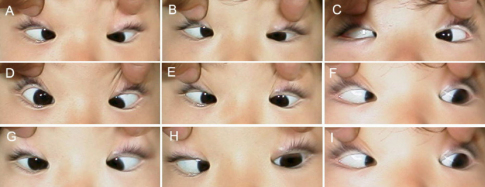
Motility photographs of individual III:1 in pedigree YT. Photos are taken in primary gaze (**E**) and in the 8 cardinal gaze positions (**A**, **B**, **C**, **D**, **F**, **G**, **H**, **I**). This individual has typical CFEOM1 features, including infraduction of the eyes in primary position, inability to elevate the eyes of midline, and ptosis (not shown).

Family YT is from Shandong Province, China. Individual II:1 is the only subject in this study without congenital ptosis. She had undergone bilateral inferior rectus recessions and superior rectus resections at age 18 years. Her eyes can elevate to the midline and the right eye is in neutral position in primary gaze post operatively (Figure 2 and Figure 3). Individual III:1 demonstrated the typical features of CFEOM1: non-progressive ptosis, infraducted globe position in primary gaze, and upgaze palsy (Figure 2 and Figure 4). At 3 years of age, she had undergone bilateral ptosis and squint surgery. Preoperative forced duction tests confirmed tight inferior recti and medial recti. Clinicopathological studies showed fibrotic extraocular muscles in the proband. Bell phenomenon was absent.

### Molecular genetics

**Figure 5:**
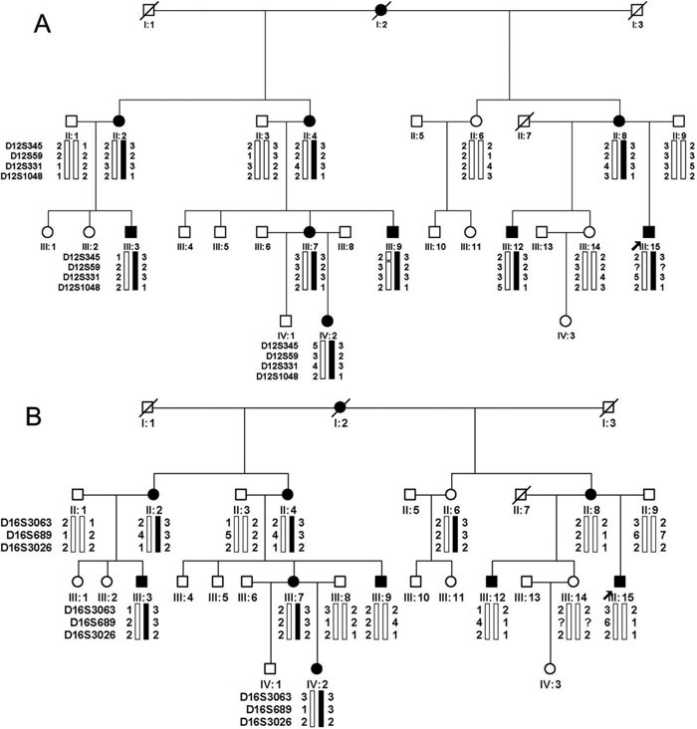
Pedigree and haplotype analysis of family XT at the FEOM1 and FEOM3 loci. Black symbols identify clinically affected individuals. Genotyping data and schematic haplotype bars for FEOM1 markers (**A**) and FEOM3 markers (**B**) are shown below the symbol for each individual. The black bars denote the potential disease-associated alleles. The white bars indicate the inheritance of non disease-associated haplotypes.

**Figure 6:**
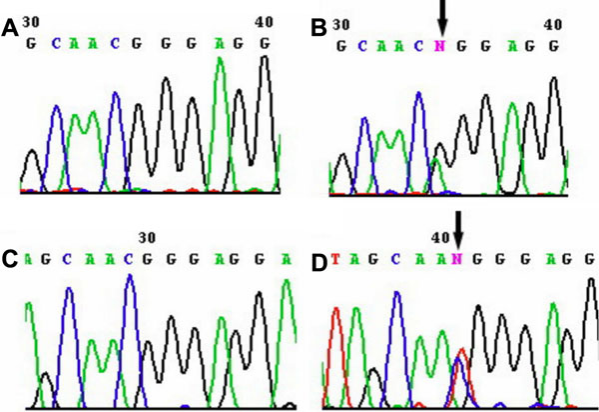
Sequence chromatographs of Family XT (**A**, **B**) and YT (**C**, **D**). The sequence of the unaffected member is normal (**A**, **C**), the affected patient with CFEOM harbor the heterozygous *KIF21A* mutation 2861G>A (**B**) and 2860C>T (**D**), respectively.

Genetic analysis revealed probable linkage of the CFEOM1 phenotype in family XT to the *KIF21A* locus with a maximum lod score of 2.24 at D12S331 (theta=0, 100% penetrance). Linkage to the FEOM3 locus was ruled-out (Table 3, Figure 5). A missense heterozygous mutation in *KIF21A* was identified in this family: 2,861G>A (R954Q; Figure 6A,B). This mutation was detected in all affected and no affected family members and not in 100 control subjects.

**Table 3:** Lod scores for the Chinese pedigree XT with CFEOM.

**Recombination fraction (θ)**									
**Marker**	**0.00**	**0.01**	**0.05**	**0.10**	**0.20**	**0.30**	**0.40**	**Z_max_**	**θ_max_**
**Family XT, 100% penetrance:**									
D12S345	1.49	1.45	1.31	1.13	0.73	0.34	0.05	1.49	0.00
D12S59	0.54	0.52	0.47	0.40	0.27	0.14	0.04	0.54	0.00
D12S331	2.24	2.20	2.02	1.79	1.28	0.75	0.26	2.24	0.00
D12S1048	2.04	2.00	1.84	1.63	1.18	0.71	0.28	2.04	0.00
D16S3063	-∞	-2.39	-1.05	-0.53	-0.12	0.02	0.04	0.04	0.40
D16S689	-∞	-3.12	-1.15	-0.41	0.14	0.27	0.21	0.27	0.30
D16S3026	-∞	-1.90	-0.61	-0.15	0.14	0.17	0.09	0.17	0.30

**Figure 7:**
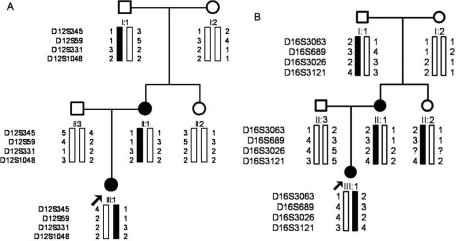
Pedigree and haplotype analysis of Family YT at the FEOM1 and FEOM3 loci. Black symbols identify clinically affected individuals. Genotyping data and schematic haplotype bars for FEOM1 markers (**A**) and FEOM3 markers (**B**) are shown below the symbol for each individual. The black bars denote the potential disease-associated alleles. The white bars indicate the inheritance of non disease-associated haplotypes.

Linkage could not be established in Family YT because only two individuals were affected. However, haplotype data were used to determine co-inheritance of the CFEOM phenotype with the FEOM1 and FEOM3 loci (Figure 7). Sequence analysis of three coding exons in *KIF21A* revealed a missense de novo heterozygous mutation (2,860C>T, R954W) in affected individuals II:1 and III:1 (Figure 6C,D). The remaining family members did not harbor the mutation, including I:1. Thus, the mutation arose de novo in II:1 on the allele she inherited from her father. Others have also reported the occurance of de novo *KIF21A* mutations on the paternal allele [13].

## Discussion

Recent studies suggest that the ptosis and primary defect in vertical gaze found in CFEOM1 patients result from aberrant development of the superior division of the oculomotor nerve (cranial nerve III) or its subnuclei [7,19]. Autopsy studies of an individual with CFEOM1 demonstrated a decrease in the number of alpha motoneurons in all oculomotor subnuclei as well as in the abducens nucleus. This finding suggests that the *KIF21A* gene product may play a more generalized role in the development of all of the ocular motor nuclei (cranial nerves III, IV, and VI). Thus, *KIF21A* may be essential for the normal development and/or axonal projection of a subset of human alpha motoneurons in the brainstem. Studies to date have provided strong evidence that the CFEOM1 phenotype results from mutations in *KIF21A* and that sporadic cases are due to de novo mutations in the same gene [20,21].

*KIF21A,* at the FEOM1 locus spans ~150 kb of genomic DNA has an open reading frame of 5,022 bp and consists of 38 exons with alternative splicing of exon 12 and exons 29–31 [6]. This gene is predicted to encode a kinesin motor protein, KIF21A, which is responsible for the transport of membranous organelles, protein complexes, and mRNAs to specific destinations within the cell in a microtubule- and ATP-dependent manner. These functions are essential for normal morphogenesis and functioning of the cell [22].

Mutational analysis of *KIF21A* in 70 CFEOM1 probands revealed a total of 12 different missense mutations [6,9,21,23-25]. Of these mutations, mutation 1067T>C (M356T) was observed in exon8, mutations 2,830G>C (E944Q), 2,839A>G (M947V), 2,840T>C(M947T) and 2,840T>G (M947R) were observed in exon 20, and mutations 2,860C>T (R954W), 2,861G>A (R954Q), 2,861G>T (R954L), 3,022G>C (A1008P) and 3,029T>C (I1010T) were observed in exon 21, mutation 84C>G (C28W)were observed in exon 2. The 2,860C>T (70%) and 2,861G>A (11%) mutations were the most commonly identified mutations in the populations of Caucasian, Hispanic, Turkish, Iranian, African, Saudi Arabian, French, Canadian, Australian, Swiss, German, Venezuelan, Italian, Chilian, Indian, English, and Taiwanese [21]. The most commonly identified *KIF21A* mutation, 2,860C>T (R954W) has subsequently been observed in families from China [26]. Ali et al. [27] demonstrated that all the CpG dinucleotides in exon 21, including the dinucleotides that harbor the two most frequently encountered *KIF21A* mutations, were methylated in genomic DNA from blood and sperm cells. The authors proposed that methylation of certain CpG dinucleotides in *KIF21A* makes them more susceptible to disease-causing mutations at specific genetic locations.

The diagnosis of CFEOM1 in family XT was based upon the observation of autosomal dominant inheritance, bilateral congenital ptosis, bilateral infraducted globe position in primary gaze, and severely restricted upgaze. Genetic analysis demonstrated that the phenotype was linked to the FEOM1 locus. A missense heterozygous mutation 2,861G>A (R954Q) was identified in this family. This mutation is the second most commonly observed *KIF21A* mutation in previously reported pedigrees and sporadic cases.

Based on the clinical examination and photographs at different ages, affected member III:1 of pedigree YT has typical CFEOM1 features with congenital non-progressive bilateral ptosis, downward eye position, inability to elevate the eyes above the horizontal midline, and aberrant eye movements. In contrast, affected member II:1 does not have ptosis or aberrant eye movements; the absence of ptosis results in a diagnosis of CFEOM3 rather than CFEOM1. Sequencing revealed that the two affected individuals in this family harbored the most commonly observed *KIF21A* mutation, 2,860C>T (R954W), that arose de novo on the paternal allele of II:1. There are previous reports of *KIF21A* mutations associated with the CFEOM3 phenotype, as well as of de novo *KIF21A* mutations, which typically occur on the paternal allele [13]. The *KIF21A* 2,860C>T mutation has also been reported in a Taiwanese pedigree [23] and a Chinese family with CFEOM3 [26].

These pedigrees support the previous reports of both inherited and de novo hot-spot *KIF21A* mutations underlying the CFEOM1 and, rarely, the CFEOM3 phenotype.
